# Meta-Analysis of Cardiovascular Risk Factors in Offspring of Preeclampsia Pregnancies

**DOI:** 10.3390/diagnostics13040812

**Published:** 2023-02-20

**Authors:** Weikai Wang, Ru Lin, Lan Yang, Yanxia Wang, Baohong Mao, Xiaoying Xu, Jing Yu

**Affiliations:** 1 The Second School of Clinical Medicine, Lanzhou University, Lanzhou 730000, China; 2 Department of PICU, Gansu Provincial Maternity and Child-Care Hospital, Lanzhou 730000, China; 3 Endoscopy Center, Gansu Provincial Maternity and Child-Care Hospital, Lanzhou 730000, China; 4 Scientific Research Center, Gansu Provincial Maternity and Child-Care Hospital, Lanzhou 730000, China; 5 Perinatal Medical Center, Gansu Provincial Maternity and Child-Care Hospital, Lanzhou 730000, China; 6 Hypertension Center, The Second Hospital of Lanzhou University, Lanzhou 730000, China

**Keywords:** preeclampsia, pregnancy offspring, cardiovascular risk factors, meta-analysis, dyslipidemia

## Abstract

This study aimed to assess cardiovascular risk factors in the offspring of preeclampsia (PE) pregnancies. PubMed, Web of Science, Ovid, and other foreign language databases, as well as SinoMed, China National Knowledge Infrastructure, Wanfang, and China Science and Technology Journal Databases, were searched. The case-control studies on cardiovascular risk factors in the offspring of PE pregnancies from 1 January 2010 to 31 December 2019 were collected. A random-effects model or a fixed-effects model was used, and RevMan 5.3 software was used for meta-analysis to determine the OR value and 95%CI of each cardiovascular risk factor. A total of 16 documents were included in this research, all of which were case-control studies, with a total of 4046 cases in the experimental group and 31,505 in the control group. The meta-analysis that was conducted demonstrated that SBP [MD = 1.51, 95%CI (1.15, 1.88)] and DBP [MD = 1.90, 95%CI (1.69, 2.10)] values in the PE pregnancy offspring group presented an elevation relative to the non-PE pregnancy offspring group. The total cholesterol value in the PE pregnancy offspring group presented an elevation relative to the non-PE pregnancy offspring group [MD = 0.11, 95%CI (0.08, 0.13)]. The low-density lipoprotein cholesterol value in the PE pregnancy offspring group was comparable to that in the non-PE pregnancy offspring group [MD = 0.01, 95%CI (−0.02, 0.05)]. The high-density lipoprotein cholesterol value in the PE pregnancy offspring group presented an elevation relative to the non-PE pregnancy offspring group [MD = 0.02, 95%CI (0.01, 0.03)]. The non-HDL cholesterol value in the PE pregnancy offspring group presented an elevation relative to the non-PE pregnancy offspring group [MD = 0.16, 95%CI (0.13, 0.19)]. The triglycerides [MD = −0.02, 95%CI (−0.03, −0.01)] and glucose [MD = −0.08, 95%CI (−0.09, −0.07)] values in the PE pregnancy offspring group presented a depletion relative to the non-PE pregnancy group. The insulin value in the PE pregnancy offspring group presented a depletion relative to the non-PE pregnancy offspring group [MD = −0.21, 95%CI (−0.32, −0.09)]. The BMI value in the PE pregnancy offspring group presented an elevation relative to the non-PE pregnancy offspring group [MD = 0.42, 95%CI (0.27, 0.57)]. In conclusion, dyslipidemia, elevated blood pressure, and increased BMI occur postpartum with PE, all of which are risk factors for cardiovascular diseases.

## 1. Introduction

Preeclampsia (PE) is a pregnancy-specific syndrome that occurs after 20 weeks of gestation and is characterized by new-onset hypertension, proteinuria, and multiple organ dysfunction [1]. The incidence of PE is 2–8% [2]. PE seriously threatens the safety of the mother and fetus [3], it accounts for 15% of maternal deaths, and it is the third leading cause of maternal death [4]. The hazards to mother and fetus during pregnancy mainly include placental abruption, cerebrovascular accidents, pulmonary edema, heart failure, liver and kidney failure, disseminated intravascular coagulation (DIC), hemolysis, elevated liver enzymes, and low platelets (HELLP) syndrome, eclampsia complicated by adult respiratory distress syndrome, fetal growth restriction (FGR), fetal distress, stillbirth, etc. According to the Report of the American College of Obstetricians and Gynecologists’ Task Force on Hypertension in Pregnancy, the current diagnostic criteria of PE include blood pressure ≥140 mmHg systolic or ≥90 mmHg diastolic on two occasions at least 4 h apart after 20 weeks of pregnancy in a woman with a previously normal blood pressure and proteinuria ≥300 mg/24 h of urine collection [5].

PE is not just a pathological pregnancy problem that accompanies pregnancy and delivery, and its postpartum risks to the mother still exist [6], including an increased risk of postpartum cardiovascular diseases and metabolic diseases [7]. Cardiovascular disease is the leading cause of morbidity and mortality in the whole world. In 2015, the World Health Organization (WHO) estimated that cardiovascular disease accounted for more than 17.7 million deaths, representing a total 31% of global deaths [8]. Cardiovascular metabolic risk (CMR) refers to a set of risk factors for cardiovascular diseases and diabetes risks, including age, race, gender, family history, overweight and obesity, abnormal glucose metabolism, abnormal lipid metabolism, elevated blood pressure, metabolic syndrome, smoking, physical inactivity, inflammation, hypercoagulability, etc. [9]. In 2011, the guidelines for reducing the risk of cardiovascular diseases in women issued by the American Heart Association (AHA) clearly listed PE as a risk factor for cardiovascular disease in women for the first time and clearly evaluated women’s cardiovascular risk from a metabolic perspective [10]. Several studies have confirmed that women who develop PE are at an increased risk of cardiovascular diseases later in life. It is reported that women with PE have about twice the risk of cardiovascular diseases, ischemic heart disease, and stroke as other women. Compared with women with normal pregnancy, women with PE are also more likely to have the risk factors of cardiovascular disease [11]. Importantly, recent emerging research also shows that the risk of cardiovascular diseases in the offspring of PE pregnancies also increases in adulthood. A research report shows that, compared with the offspring of normal pregnancy, the risk of stroke in the offspring of PE pregnancy is about twice as high [12]. Another study investigated the risk factors of cardiovascular diseases in the offspring of PE pregnancy and found that the young offspring of PE pregnancy had higher blood pressure and body mass index (BMI) [13]. To date, the cardiometabolic risks of PE include postpartum hypertension, diabetes and abnormal glucose metabolism, dyslipidemia, and metabolic syndrome. Identifying cardiovascular risk factors in the offspring of PE pregnancies and implementing prevention and early intervention is of great significance for reducing cardiovascular morbidity and improving perinatal outcomes in offspring of PE pregnancies.

This study aimed to identify which cardiovascular risk factors the offspring of PE pregnancies exhibit and may be utilized for screening for primary prevention of cardiovascular disease in the offspring of PE pregnancies. We focused on the most common cardiovascular risk factors, such as glucose, insulin, triglycerides, total cholesterol, BMI, low-density lipoprotein cholesterol, high-density lipoprotein (HDL) cholesterol, non-HDL cholesterol, and blood pressure. This research conducted a systematic review and meta-analysis of independent studies published from 1 January 2010 to 31 December 2019, with the aim of screening out the cardiovascular risk factors and association strengths of offspring of PE pregnancies, providing evidence for the prevention and intervention of cardiovascular diseases in the offspring of PE pregnancies.

## 2. Materials and Methods

### 2.1. Literature Retrieval Strategies

**Figure 1:**
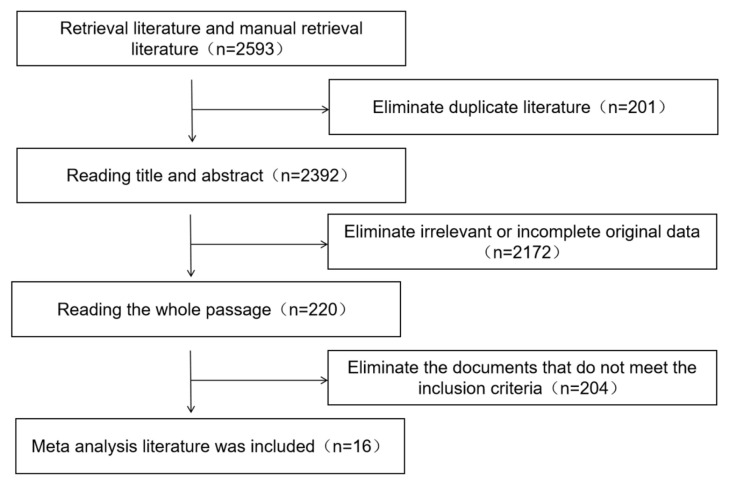
The flowchart of the literature retrieval and screening.

Foreign language databases such as PubMed, Web of Science, Ovid, etc., as well as SinoMed, China National Knowledge Infrastructure (CNKI), Wanfang, and China Science and Technology Journal Database (CQVIP), were searched. From the English literature, case-control studies on cardiovascular risk factors in offspring of PE pregnancies from 1 January 2010 to 31 December 2019 were collected. The English retrieval terms were “Cardiovascular”, “risk factor”, “influence factor”, and “preeclampsia pregnancy”. The method of combining subject headings and keywords was used for retrieval under the language limited to English. For a more comprehensive retrieval of the desired literature, after the databases was searched, the references of the included literature were manually searched. The literature screening process is shown in Figure 1.

### 2.2. Inclusion and Exclusion Criteria

Inclusion criteria: (i) The type of study was a case-control study. (ii) The definitions and quantification of risk-factor variables were basically the same in all studies. (iii) The risk indicators (OR value and 95%CI) of the research factors could be provided or further calculated from the data. (iv) When calculating the risk indicators of related factors, univariate and multivariate methods were used for the analysis. (v) At least one risk factor was included.

Exclusion criteria: (i) An evaluation was performed according to the Cochrane-recommended non-randomized controlled study systematic review tool—NOS—including the selection of study subjects, comparability between groups, and outcome measurement or exposure factor measurement, with a total score of 9 points. Works in from the literature that were duplicate reports, were of low quality, had too little reported information, and were unusable articles were excluded. (ii) The risk factors studied were laboratory-related indicators.

### 2.3. Literature Inclusion and Data Extraction

Methods of literature inclusion: The literature works were included by one researcher according to the inclusion criteria and reviewed by another researcher. When the researchers disagreed, the decision was made through discussion.

Data extraction: Data were extracted by one researcher according to a predesigned table, including general characteristics of research, type of research, research subjects, research factors, and research results, and they were reviewed by another researcher. When the two researchers disagreed, the decision was made through discussion.

### 2.4. Statistical Analysis

All processes of the meta-analysis were carried out by using RevMan5.3 software, the final-effects indicators were measured by mean ± standard deviation (mean ± SD), and the heterogeneity was assessed by using Q test and I^2^. If *p* ≥ 0.05 and I^2^ ≤ 50%, it indicated no statistical heterogeneity or small heterogeneity among the study results, and a fixed-effects model could be used for the meta-analysis; if *p* < 0.05 and I^2^ > 50%, it indicated statistical heterogeneity among the study results, and a random-effects model could be used for the meta-analysis. Evidence-based medicine suggested the greater stability of the random-effects model than the fixed-effects model. If the 95%CI of the combined OR value did not include 1.0, the OR value presented statistical significance at 0.05; if the 95%CI of the combined MD value included 0, the OR value presented no statistical difference at 0.05.

## 3. Results

### 3.1. Basic Characteristics of the Literature

A total of 16 articles were included in this research, all of which were case-control studies, with a total of 4046 cases in the experimental group and 31,505 in the control group. The quality evaluation of the included literature is shown in Table 1, and basic information is given in Table 2.

### 3.2. Results of Meta-Analysis

#### 3.2.1. Systolic Blood Pressure

**Figure 2:**
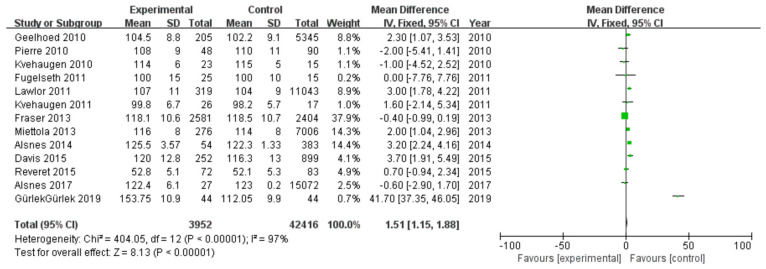
Meta-analysis forest map for systolic blood pressure of offspring of PE pregnancies. Geelhoed 2010, [17]; Pierre 2010, [18]; Kvehaugen 2010, [16]; Fugelseth 2011, [19]; Lawlor 2011, [20]; Kvehaugen 2011, [21]; Fraser 2013, [24]; Miettola 2013, [23]; Alsnes 2014, [25]; Davis 2015, [26]; Reveret 2015, [27]; Alsnes 2017, [25]; Gürlek 2019 [29].

Among 16 included studies, 13 studies investigated SBP data of patients, including 3952 patients in the experimental group and 42,416 in the control group, with data heterogeneity (I^2^ = 97%) (Figure 2). The analysis showed that the SBP value in the PE pregnancy offspring group presented an elevation relative to the non-PE pregnancy offspring group [MD = 1.51, 95%CI (1.15, 1.88), *p* < 0.00001], with statistical significance (Figure 2).

#### 3.2.2. Diastolic Blood Pressure

**Figure 3:**
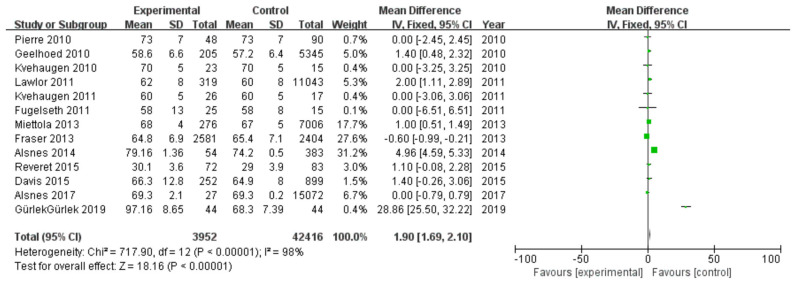
Meta-analysis forest map for diastolic blood pressure of offspring of PE pregnancies. Pierre 2010, [18]; Geelhoed 2010, [17]; Kvehaugen 2010, [16]; Lawlor 2011, [20]; Kvehaugen 2011, [21]; Fugelseth 2011, [19]; Miettola 2013, [23]; Fraser 2013, [24]; Alsnes 2014, [25]; Reveret 2015, [27]; Davis 2015, [26]; Alsnes 2017, [25]; Gürlek 2019 [29].

Among 16 included studies, 13 studies investigated the DBP data of patients, including 3952 patients in the experimental group and 42,416 in the control group, with data heterogeneity (I^2^ = 98%) (Figure 3). The analysis showed that the DBP value in the PE pregnancy offspring group presented an elevation relative to the non-PE pregnancy offspring group [MD = 1.90, 95%CI (1.69, 2.10), *p* < 0.00001], with statistical significance (Figure 3).

#### 3.2.3. Total Cholesterol

**Figure 4:**
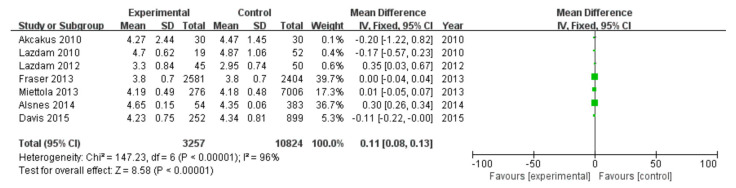
Meta-analysis forest map for total cholesterol of offspring of PE pregnancies. Akcakus 2010 [14]; Lazdam 2010 [15]; Lazdam 2012 [22]; Fraser 2013, [24]; Miettola 2013, [23]; Alsnes 2014, [25]; Davis 2015, [26].

Among 16 included studies, 7 studies evaluated the total cholesterol data of patients, including 3257 patients in the experimental group and 10,824 in the control group, with data heterogeneity (I^2^ = 96%) (Figure 4). The analysis showed that the total cholesterol value in the PE pregnancy offspring group presented an elevation relative to the non-PE pregnancy offspring group [MD = 0.11, 95%CI (0.08, 0.13), *p* < 0.00001], with statistical significance (Figure 4).

#### 3.2.4. Low-Density Lipoprotein Cholesterol

**Figure 5:**
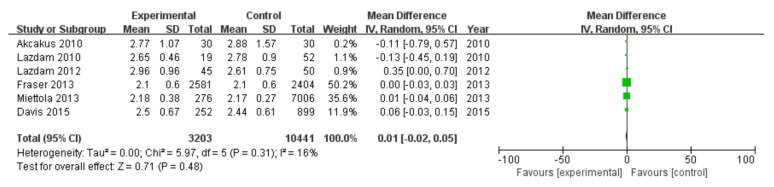
Meta-analysis forest map for low-density lipoprotein cholesterol of offspring of PE pregnancies. Akcakus 2010 [14]; Lazdam 2010 [15]; Lazdam 2012 [22]; Fraser 2013, [24]; Miettola 2013, [23]; Davis 2015, [26].

Among 16 included studies, 6 studies investigated low-density lipoprotein cholesterol data of patients, including 3203 patients in the experimental group and 10,441 in the control group, without data heterogeneity (I^2^ = 16%) (Figure 5). The analysis showed that the low-density lipoprotein cholesterol value in the PE pregnancy offspring group was comparable to that in the non-PE pregnancy offspring group [MD = 0.01, 95%CI (−0.02, 0.05), *p* = 0.48], without statistical significance (Figure 5).

#### 3.2.5. High-Density Lipoprotein Cholesterol

**Figure 6:**
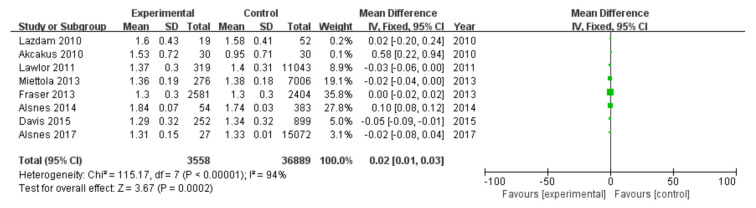
Meta-analysis forest map for high-density lipoprotein cholesterol of offspring of PE pregnancies. Lazdam 2010 [15]; Akcakus 2010 [14]; Lawlor 2011, [20]; Miettola 2013, [23]; Fraser 2013, [24]; Alsnes 2014, [25]; Davis 2015, [26]; Alsnes 2017, [28].

Among 16 included studies, 8 studies investigated high-density lipoprotein cholesterol data of patients, including 3558 patients in the experimental group and 36,889 in the control group, with data heterogeneity (I^2^ = 94%) (Figure 6). The analysis showed that the high-density lipoprotein cholesterol value in the PE pregnancy offspring group presented an elevation relative to the non-PE pregnancy offspring group [MD = 0.02, 95%CI (0.01, 0.03), *p* = 0.0002], with statistical significance (Figure 6).

#### 3.2.6. Non-HDL Cholesterol

**Figure 7:**
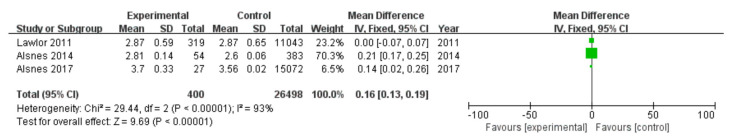
Meta-analysis forest map for non-HDL cholesterol of offspring of PE pregnancies. Lawlor 2011, [20]; Alsnes 2014, [25]; Alsnes 2017, [28].

Among 16 included studies, 3 studies investigated non-HDL cholesterol data of patients, including 400 patients in the experimental group and 26,498 in the control group, with data heterogeneity (I^2^ = 93%) (Figure 7). The analysis showed that the non-HDL cholesterol value in the PE pregnancy offspring group presented an elevation relative to the non-PE pregnancy offspring group [MD = 0.16, 95%CI (0.13, 0.19), *p* < 0.00001], with statistical significance (Figure 7).

#### 3.2.7. Triglycerides

**Figure 8:**
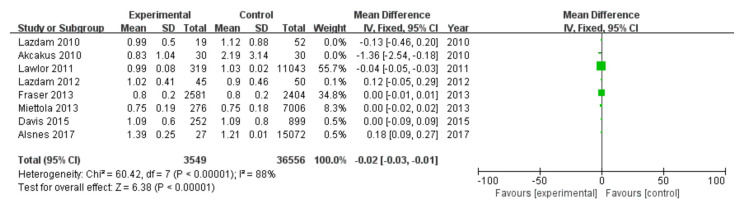
Meta-analysis forest map for triglycerides of offspring of PE pregnancies. Lazdam 2010 [15]; Akcakus 2010 [14]; Lawlor 2011, [20]; Lazdam 2012 [22]; Fraser 2013 [24]; Miettola 2013, [23]; Davis 2015, [26]; Alsnes 2017, [28].

Among 16 included studies, 8 studies investigated the triglycerides data of patients, including 3549 patients in the experimental group and 36,556 in the control group, with data heterogeneity (I^2^ = 88%) (Figure 8). The analysis showed that the triglycerides value in the PE pregnancy offspring group presented a depletion relative to the non-PE pregnancy offspring group [MD = −0.02, 95%CI (−0.03, −0.01), *p* < 0.00001], with statistical significance (Figure 8).

#### 3.2.8. Glucose

**Figure 9:**
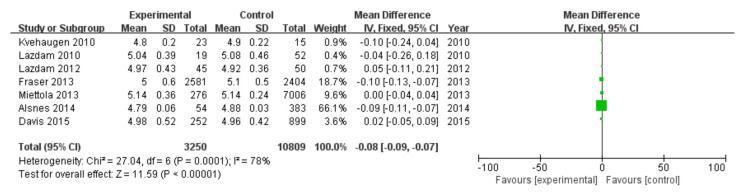
Meta-analysis forest map for glucose of offspring of PE pregnancies. Kvehaugen 2010, [16]; Lazdam 2010 [15]; Lazdam 2012 [22]; Fraser 2013 [24]; Miettola 2013, [23]; Alsnes 2014, [25]; Davis 2015, [26].

Among 16 included studies, 7 studies investigated the glucose data of patients, including 3250 patients in the experimental group and 10,809 in the control group, with data heterogeneity (I^2^ = 78%) (Figure 9). The analysis showed that the glucose value in the PE pregnancy offspring group presented a depletion relative to the non-PE pregnancy offspring group [MD = −0.08, 95%CI (−0.09, −0.07), *p* < 0.00001], with statistical significance (Figure 9).

#### 3.2.9. Insulin

**Figure 10:**
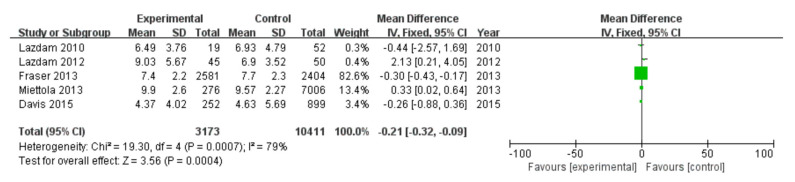
Meta-analysis forest map for insulin of offspring of PE pregnancies. Lazdam 2010 [15]; Lazdam 2012 [22]; Fraser 2013 [24]; Miettola 2013, [23]; Davis 2015, [26].

Among 16 included studies, 5 studies investigated the insulin data of patients, including 3173 patients in the experimental group and 10,411 in the control group, with data heterogeneity (I^2^ = 79%) (Figure 10). The analysis showed that the insulin value in the PE pregnancy offspring group presented a depletion relative to the non-PE pregnancy offspring group [MD = −0.21, 95%CI (−0.32, −0.09), *p* = 0.0004], with statistical significance (Figure 10).

#### 3.2.10. BMI

**Figure 11:**
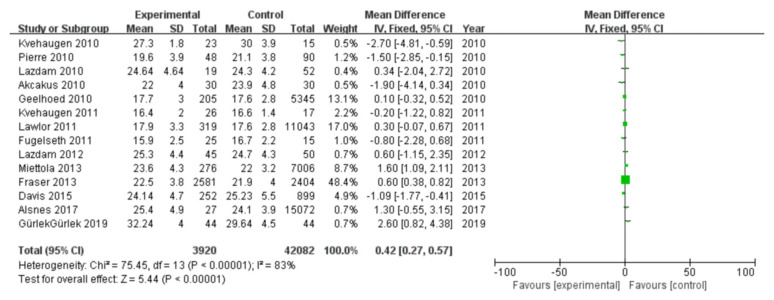
Meta-analysis forest map for BMI of offspring of PE pregnancies. Kvehaugen 2010, [16]; Pierre 2010, [18]; Lazdam 2010 [15]; Akcakus 2010 [14]; Geelhoed 2010, [17]; Kvehaugen 2011, [21]; Lawlor 2011, [20]; Fugelseth 2011, [19]; Lazdam 2012 [22]; Miettola 2013, [23]; Fraser 2013, [24]; Davis 2015, [26]; Alsnes 2017, [25]; Gürlek 2019 [29].

Among 16 included studies, 14 studies investigated the BMI data of patients, including 3920 patients in the case group and 42,082 in the control group, with data heterogeneity (I^2^ = 83%) (Figure 11). The analysis showed that the BMI value in the PE pregnancy offspring group presented an elevation relative to the non-PE pregnancy offspring group [MD = 0.42, 95%CI (0.27, 0.57), *p* < 0.00001], with statistical significance (Figure 11).

### 3.3. Analysis of Publication Bias

**Figure 12:**
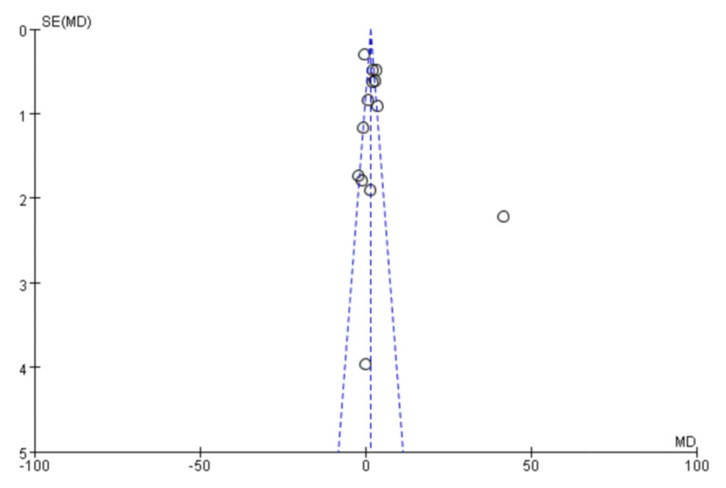
Funnel plot.

The accuracy of the conclusions of the meta-analysis largely depends on the completeness of included studies, which can be measured by reporting bias. Publication bias is one of the reporting biases that has received the most attention. By drawing a funnel plot, we found that one of the included works from the literature was outside the confidence interval, and the rest were within the confidence interval. Moreover, the distribution was relatively clustered, indicating that there was a certain publication bias, and the publication bias was not large (Figure 12).

## 4. Discussion

PE is a pregnancy-specific syndrome that occurs after 20 weeks of gestation and is characterized by new-onset hypertension, proteinuria, and multiple organ dysfunction [30]. The incidence of PE ranges from 2% to 8% [31]. PE seriously threatens the safety of the mother and fetus [32], and it is the third leading cause of maternal death [33]. The hazards to the mother and fetus during pregnancy mainly include placental abruption, cerebrovascular accidents, pulmonary edema, heart failure, liver and kidney failure, DIC, HELLP syndrome, eclampsia complicated by adult respiratory distress syndrome, FGR, fetal distress, stillbirth, etc. Furthermore, PE is not just a pathological pregnancy problem that accompanies pregnancy and delivery; its postpartum risks to the mother still exist, including an increased risk of postpartum cardiovascular diseases and metabolic diseases [34]. CMR refers to a set of risk factors for cardiovascular diseases and diabetes risks, including age, race, gender, family history, overweight and obesity, abnormal glucose metabolism, abnormal lipid metabolism, elevated blood pressure, metabolic syndrome, smoking, physical inactivity, inflammation, hypercoagulability, etc. [33]. In 2011, the guidelines for reducing the risk of cardiovascular diseases in women issued by the AHA clearly listed PE as a risk factor for cardiovascular disease in women for the first time. National guidance in England suggests that women who suffer from PE should be informed of the elevated risk of high blood pressure and some other related complications [35]. Moreover, in the US, the AHA suggests that women with a history of pregnancy-induced hypertension should be referred to estimate cardiovascular risk factors [36]. A systematic review and meta-analysis showed that, in comparison to women with single PE and subsequent uncomplicated pregnancy, women with recurrent PE had a three-fold increase in heart-failure risk, a two- to three-fold increase in hypertension risk, and an almost two-fold increase in overall cardiovascular disease risk [37]. The only long-term mortality follow-up study reported so far showed a 1.9-fold increased risk of stroke mortality in the offspring of PE pregnancies [12]. The occurrence and development of PE are inextricably linked with metabolism. A large number of studies have revealed that the metabolic disorder of PE persists until postpartum [38], and metabolic factors may accompany the whole process of disease development in PE patients. Metabolic factors associated with postpartum CMR will provide evidence-based medical proof for the prevention and blockade of cardiovascular disease and metabolic diseases in women.

Herein, the meta-analysis demonstrated that the SBP value in the PE pregnancy offspring group presented an elevation relative to the non-PE pregnancy offspring group [MD = 1.51, 95%CI (1.15, 1.88), *p* < 0.00001]. The DBP value in the PE pregnancy offspring group presented an elevation relative to the non-PE pregnancy offspring group [MD = 1.90, 95%CI (1.69, 2.10), *p* < 0.00001]. The total cholesterol value in the PE pregnancy offspring group presented an elevation relative to the non-PE pregnancy offspring group [MD = 0.11, 95%CI (0.08, 0.13), *p* < 0.00001]. The low-density lipoprotein cholesterol value in the PE pregnancy offspring group was comparable to that in the non-PE pregnancy offspring group [MD = 0.01, 95%CI (−0.02, 0.05), *p* = 0.48]. The high-density lipoprotein cholesterol value in the PE pregnancy offspring group presented an elevation relative to the non-PE pregnancy offspring group [MD = 0.02, 95%CI (0.01, 0.03), *p* = 0.0002]. The non-HDL cholesterol value in the PE pregnancy offspring group presented an elevation relative to the non-PE pregnancy offspring group [MD = 0.16, 95%CI (0.13, 0.19), *p* < 0.00001]. The triglycerides value in the PE pregnancy offspring group presented a depletion relative to the non-PE pregnancy group [MD = −0.02, 95%CI (−0.03, −0.01), *p* < 0.00001]. The glucose value in the PE pregnancy offspring group presented a depletion relative to the non-PE pregnancy offspring group [MD = −0.08, 95%CI (−0.09, −0.07), *p* < 0.00001]. The insulin value in the PE pregnancy offspring group presented a depletion relative to the non-PE pregnancy offspring group [MD = −0.21, 95%CI (−0.32, −0.09), *p* = 0.0004]. The BMI value in the PE pregnancy offspring group presented an elevation relative to the non-PE pregnancy offspring group [MD = 0.42, 95 %CI (0.27, 0.57), *p* < 0.00001]. It was suggested that postpartum PE patients have persistently elevated blood pressure; elevated total cholesterol; decreased fasting blood glucose, triglycerides, and insulin; elevated high-density lipoprotein; and decreased low-density lipoprotein and still have obvious insulin resistance and metabolic abnormalities.

Previously, metabolic changes amplified during pregnancy with PE persisted from 24 to 48 h postpartum [39] to 3 months postpartum [40]. Thus, postpartum metabolic abnormalities are very likely to be a continuation of metabolic abnormalities during pregnancy. An elevated insulin level is an indirect sign of insulin resistance. Insulin resistance is closely related to metabolic syndrome and leads to vascular endothelial dysfunction, dyslipidemia, hypertension, and vascular inflammation, all of which promote the development of cardiovascular disease [41]. PE patients had significant postpartum insulin resistance relative to normal controls [42], similar to our findings. Metabolic syndrome has been revealed to exert the crucial function in the pathophysiology of gestational hypertension and PE and might be the latent mechanism linking PE in pregnancy and cardiovascular disease. Girouard J et al. focused on evaluating postpartum-related metabolic changes and found that, relative to the normal pregnancy group, the postpartum body weight, LDL, lipoprotein B/lipoprotein A1, homocysteine, leptin, and insulin levels in the hypertensive disorder complicating pregnancy (HDCP) group presented a marked elevation, suggesting that the levels of various metabolic indicators of HDCP in the first 10 years after delivery were still very high (*p* < 0.004) [42], and this supported our findings. A prospective cohort study revealed that postpartum PE patients develop dyslipidemia, elevated blood pressure, and elevated BMI, which are risk factors for cardiovascular diseases. They also noted that postpartum blood pressure elevations were more pronounced in patients with recurrent PE than in patients with first PE. Postpartum systolic blood pressure was, on average, 27 mm Hg higher (95%CI: 18–37 mm Hg), and diastolic blood pressure was, on average, 12 mm Hg higher (95%CI: 5–19 mm Hg) in patients with hypertension in three consecutive pregnancies than in patients with hypertension in only one pregnancy [43]. Furthermore, a meta-analysis of 53,029 individuals of whom 1599 were exposed to PE in utero indicates a 5.17 mm Hg greater systolic blood pressure among those exposed to PE in comparison of controls, and the meta-analyses of 52,993 individuals, of whom 1583 were exposed to PE in utero, indicates a 4.06 mm Hg greater diastolic blood pressure among those exposed to PE in comparison to the controls [44]. The BMI is an anthropometric index that is utilized as a surrogate marker for fat mass and for classifying obesity. BMI and total adiposity are positively correlated with cardiometabolic disease risk at the population level. A study reported a 0.62 kg/m^2^ increase in BMI among offspring of PE pregnancies compared with controls (39,473 individuals; 1062 exposed to PE) [13].

Studies have confirmed some cardioprotective strategies of PE patients during pregnancy. SGLT2 inhibitors are reported to reduce blood pressure and proteinuria, the typical clinical manifestations of PE [45,46]. Different gliflozins (SGLT2 inhibitors) can play a cardioprotective role, reduce cardiovascular death, and treat heart failure [47]. Since November 2020, dapagliflozin has been approved for the first time as an SGLT2 inhibitor for the treatment of patients with heart failure. Empagliflozin lowered by 32%, 38%, and 35% the risk of all-cause mortality, cardiovascular mortality, and hospitalization due to worsening heart failure in Type 2 Diabetes Mellitus patients [48]. The CANVAS program has indicated that canagliflozin lowers the risk of cardiovascular death by 14% and the risk of heart failure hospitalization by 33% [49]. Ruonan Zhai et al. revealed that oral empagliflozin can reduce high systolic blood pressure and proteinuria and improve kidney histopathology, thereby improving PE without affecting fetal outcomes [50]. Statins are used for the treatment and prevention of cardiovascular diseases through lipid-lowering therapy [51]. The effect of pravastatin during pregnancy has been demonstrated in many rodent models of PE [52]. Studies have shown that pravastatin treatment can significantly reduce the maternal sFlt-1 level, lower blood pressure, and improve vascular conditions [53,54]. Aspirin is the most commonly used treatment to prevent cardiovascular complications [55]. Studies have shown that aspirin therapy initiated at ≥16 weeks of gestation is associated with a 50% reduction in PE in preterm infants with a dose-dependent effect [56]. A clinical trial involving 1776 patients with low early placental growth factor level reported that a daily dose of 150 mg aspirin could reduce PE by 62% [57]. On the other hand, nutraceuticals have proved to be of great benefit in combating the progress of cardiovascular disease [58]. The nano drug delivery system has shown remarkable results in delivering nutraceuticals in various diseases, including cancer, neurodegenerative diseases, cardiovascular diseases, etc. [59]. Common nutraceuticals in cardiovascular diseases include resveratrol, vitamin D, quercetin, curcumin, flavanol, etc. Resveratrol is a polyphenolic compound from the stilbene group and is mainly a component of red wines. It has been shown to have cardioprotective effects due to its anti-inflammatory and antioxidant properties [60]. Reports have indicated that resveratrol exerts a protective function in animals with dyslipidemia and insulin resistance, and it can reduce cardiac hypertrophy and systolic dysfunction [61]. Quercetin is a flavonoid that improves lipid metabolism, vascular function, blood pressure, and glucose metabolism, and it is thought to reduce or prevent the progression of cardiovascular disease [62]. One study showed that 730 mg of quercetin given daily for four weeks reduces systolic and diastolic blood pressure in patients with stage 1 hypertension [63]. Vitamin D deficiency has been associated with the development of cardiovascular disease, microbial infections, or tumor development [64]. In a cohort study performed on 13,806 pregnant women, maternal vitamin D deficiency was intensely related to an increased risk for PE [65]. Studies have shown that low maternal vitamin D levels are associated with a roughly twofold increase in the prevalence of congenital heart defects in offspring [66]. Vitamin D supplementation has been proved to enhance the effect of nifedipine in the treatment of PE [67]. Nanoparticles can be absorbed on the plasma membrane through endocytosis and non-endocytosis, and they can be transferred from passive diffusion to active transport [68]. Nanocarriers involved in drug delivery can increase the water solubility of insoluble drugs, thus preventing the degradation and inactivation of active ingredients [69]. Hyaluronic acid (HA) is a natural mucopolysaccharide and is the main constituent of the extracellular matrix, which exerts a crucial role in cell growth and in maintaining the structural stability of tissue [70]. Importantly, because HA is biocompatible, non-immunogenic, non-toxic, biodegradable, chemically modifiable, highly hydrophilic, and can absorb water to produce viscoelastic gel, it has been widely studied as a drug delivery system [71]. The hydrophilic shell of hyaluronic acid granules extends its circulation time in the blood, which can increase the probability of reaching the treatment site after systemic administration [72]. The development of hyaluronic-acid-based nanomedicines can help improve the oral bioavailability of cardioprotective natural molecules such as quercetin, resveratrol, and vitamin D. This may be an effective treatment strategy to reduce cardiovascular risk factors in the offspring of PE patients.

Herein, the risk factors of postpartum cardiovascular risk in PE patients were mainly metabolic factors in the fully developed stage of the disease. Though prenatal basic BMI, blood pressure and other factors were also considered, prenatal metabolic factors and other possible factors were not considered, and the risk factors for postpartum risk of PE were not fully understood. In our research, relevant documents were collected as comprehensively as possible, and the literature was screened strictly according to the inclusion and exclusion criteria, as well as literature quality, to avoid retrieval bias to the greatest extent. Nevertheless, some documents can only be discarded due to different effect indicators or the inability to calculate the original data. Additionally, language bias, publication bias, and reporting bias may all have an impact on the results of this research. Due to the limitation of the type of study, this study only searched the published literature, and the existence of unpublished studies that may affect the results of this study cannot be excluded.

## 5. Conclusions

PE, as a critical illness in obstetrics, can achieve early prevention, early diagnosis, and early intervention for high-risk groups by recognizing high-risk factors, screening high-risk groups, and avoiding exposure to some cardiovascular risk factors in early pregnancy, thereby reducing the incidence of PE in pregnancy and improving the perinatal outcomes of mothers and babies. There is a need to improve obstetricians’ awareness of the risk and risk factors for PE and to strengthen pre-pregnancy and pregnancy care.

## Figures and Tables

**Table 1:** Quality evaluation of included studies.

Author	Year of Publication	Selection of Research Subjects (4 Points)	Comparability between Groups (2 Points)	Outcome Measurement/Exposure Factor Measurement (3 Points)	Total Scores
Mustafa Akcakus [14]	2010	3	2	3	8
Merzaka Lazdam [15]	2010	3	2	2	7
ANNE STINE Kvehaugen [16]	2010	4	2	3	9
JJ Miranda Geelhoed [17]	2010	3	1	3	7
Pierre-Yves Jayet, MD [18]	2010	4	1	3	8
Drude Fugelseth [19]	2011	4	2	3	9
Debbie Anne Lawlor [20]	2011	3	1	2	6
Anne Stine Kvehaugen [21]	2011	4	1	3	8
Merzaka Lazdam [22]	2012	4	2	3	9
Satu Miettola [23]	2013	4	1	1	6
Abigail Fraser [24]	2013	4	2	2	8
Ingvild V. Alsnes [25]	2014	4	1	3	8
Esther F Davis [26]	2015	3	2	3	8
M Reveret [27]	2015	3	1	3	7
Ingvild V. Alsnes [28]	2017	4	1	2	7
Beril Gürlek [29]	2019	4	2	3	9

**Table 2:** Basic information of included studies.

Author	Year	Type of Study	N		The Studied Risk Factors
			Experimental Group	Control Group	
Mustafa Akcakus [14]	2010	Case-control	30	30	c,d,e,g,i
Merzaka Lazdam [15]	2010	Case-control	19	52	c,d,e,f,g,h,i,j
ANNE STINE Kvehaugen [16]	2010	Case-control	23	15	a,b,h,j
JJ Miranda Geelhoed [17]	2010	Case-control	205	5345	a,b,j
Pierre-Yves Jayet, MD [18]	2010	Case-control	48	90	a,b,j
Drude Fugelseth [19]	2011	Case-control	25	15	a,b,j
Debbie Anne Lawlor [20]	2011	Case-control	319	11,043	a,b,e,f,g,j
Anne Stine Kvehaugen [21]	2011	Case-control	26	17	a,b,j
Merzaka Lazdam [22]	2012	Case-control	45	50	c,d,g,h,i,j
Satu Miettola [23]	2013	Case-control	276	7006	a,b,c,d,e,g,h,i,j
Abigail Fraser [24]	2013	Case-control	2581	2404	a,b,c,d,e,f,g,i,j
Ingvild V. Alsnes [25]	2014	Case-control	54	383	a,b,c,e,f,h
Esther F Davis [26]	2015	Case-control	252	899	a,b,c,d,e,g,h,i,j
M Reveret [27]	2015	Case-control	72	83	a,b
Ingvild V. Alsnes [28]	2017	Case-control	27	15,072	a,b,c,e,f,h
Beril Gürlek [29]	2019	Case-control	44	44	a,b,j

Note: a, systolic blood pressure; b, diastolic blood pressure; c, total cholesterol; d, low-density lipoprotein cholesterol; e, high-density lipoprotein cholesterol; f, non-HDL cholesterol; g, triglycerides; h, glucose; i, insulin; j, BMI.

## Data Availability

Not applicable.
